# Transformation of Low-Grade Mucinous Neoplasm of the Appendix With Pseudomyxoma Peritonei to High-Grade Sarcomatoid Carcinoma

**DOI:** 10.14740/jocmr2178w

**Published:** 2015-05-08

**Authors:** Yu-Nung Chen, Jie-Jen Lee, Shih-Ping Cheng, Chung-Hsin Tsai

**Affiliations:** a Department of Surgery, MacKay Memorial Hospital and Mackay Medical College, Taipei, Taiwan; b Mackay Junior College of Medicine, Nursing, and Management, Taipei, Taiwan

**Keywords:** Dedifferentiation, Pseudomyxoma peritonei, Recurrence

## Abstract

A 66-year-old man initially underwent appendectomy and cytoreductive surgery for a low-grade appendiceal mucinous neoplasm with pseudomyxoma peritonei. One and a half years later, multiple disseminated lesions developed in rectus abdominis muscle and peritoneal cavity. Biopsy showed histopathological transformation to sarcomatoid carcinoma. This case illustrates that evolution of low-grade pseudomyxoma peritonei to high-grade carcinoma truly develops in some patients. The development of this dedifferentiation appears associated with aggressive behavior and poor clinical outcome.

## Introduction

Pseudomyxoma peritonei is a rare peritoneal disseminated disease usually arising from a primary appendiceal mucinous epithelial neoplasm [1]. It is characterized by peritoneal deposits of adenomucinous tumor cells producing a progressive amount of intraperitoneal mucinous ascites. Aggressive cytoreductive surgery with peritonectomy procedures combined with perioperative intraperitoneal chemotherapy can be curative, leading to long-term survival [2]. However, recurrent or progressive disease following treatment is not uncommon and may require repeated cytoreductive surgery that is usually less successful. Tumor biology is a major determinant of prognosis in recurrent or progressive pseudomyxoma peritonei [3].

Pseudomyxoma peritonei of appendiceal origin could be classified as low-grade and high-grade, given that high-grade tumor conferred a poor outcome [4]. During recurrence or progression, dedifferentiation has been reported and was shown to have a significant negative impact on survival [5]. Nonetheless, the real occurrence of dedifferentiation is controversial. It is argued that the discordant pathological findings could be a result of inadequate sampling, tumor heterogeneity, interobserver variations, and lack of uniformity in classification [6]. Here, we report an unusual case of low-grade mucinous neoplasm of the appendix associated with sarcomatoid transformation during recurrence.

## Case Report

**Figure 1:**
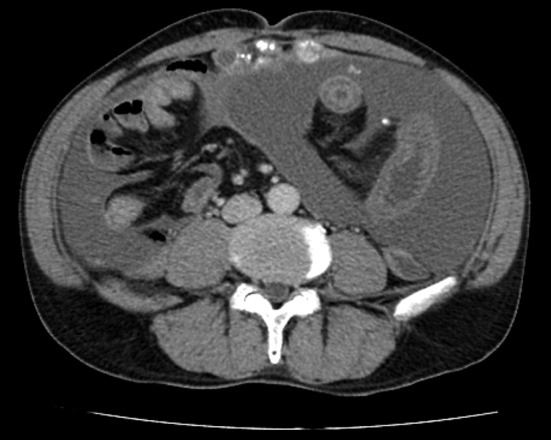
Abdominal computed tomographic scan before the initial operation showing massive ascites and peritoneal thickening with coarse calcifications.

**Figure 2:**
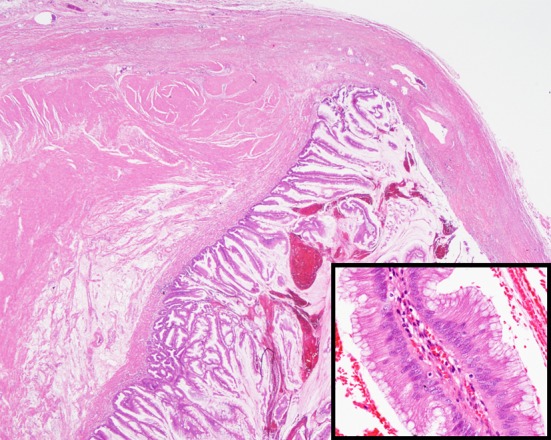
Microscopic appearance of the appendiceal tumor showing tall columnar epithelium with abundant extracellular mucin involving the submucosa and muscularis propria. Epithelium had no cytologic atypia or mitosis. The lumen was filled with pus and mucoid material (hematoxylin-eosin stain, original magnification, × 10; inset, × 200).

A 66-year-old man with unremarkable past medical history presented with dull abdominal pain lasting 5 days. He complained of nausea and vomiting without changes in bowel habit. Physical examination revealed diffuse tenderness and abdominal distention in the presence of shifting dullness. There was slight guarding of the abdominal wall without rebound tenderness. Laboratory data were within normal limits. Computed tomographic (CT) scan showed dilation of small bowel loops, large amount of ascites, and peritoneal thickening with coarse calcifications (Fig. 1). A jelly-like mucinous material was obtained on ultrasonography-guided abdominal paracentesis. The cytological examination was positive for malignant cells. Exploratory laparotomy revealed a ruptured mucinous lesion of the appendix with gelatinous pseudomyxoma peritonei. The patient underwent appendectomy and aggressive cytoreductive surgery with no residual macroscopic tumor (R1 cytoreduction). Pathological examination confirmed the diagnosis of low-grade appendiceal mucinous neoplasm (Fig. 2). His postoperative recovery was uneventful. The patient declined further adjuvant chemotherapy.

**Figure 3:**
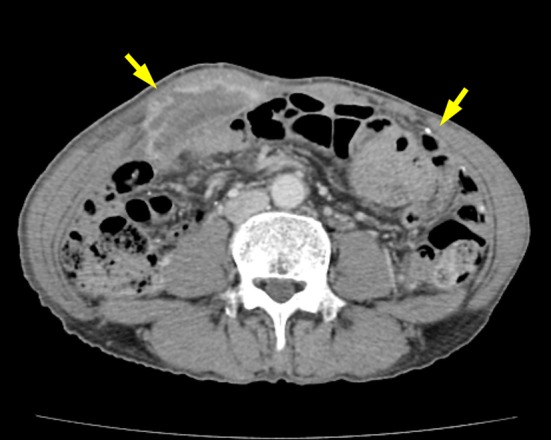
Abdominal computed tomographic scan after disease recurrence showing rim-enhanced lesions with calcification over rectus abdominis muscle and peritoneum (arrows).

**Figure 4:**
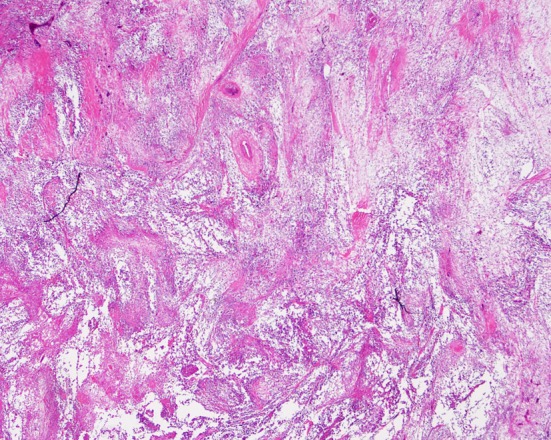
Biopsy of the recurrent lesions showing spindling and epithelioid pleomorphic tumor cells (hematoxylin-eosin stain, original magnification, × 10).

One and a half years later, the patient manifested a firm, non-tender abdominal wall mass. The abdominal CT scan revealed several rim-enhanced lesions with calcification over peritoneum, rectus abdominis muscle and mesentery (Fig. 3). Intraperitoneal fluid was not seen. A relaprotomy with debulking intent was performed. At exploration, carcinomatosis with a dominant tumor, measuring 8.6 × 6.7 × 2.5 cm, at the left upper quadrant of the abdomen was noted. Biopsy from the peritoneal cavity, mesenteric soft tissue, and part of the tumor-encased intestinal wall showed sarcomatoid carcinoma (Fig. 4). The patient died of disease 1 month after the second operation. No autopsy was performed.

## Discussion

Pseudomyxoma peritonei is a rare but intriguing disease characterized by wide spread of mucinous ascites with peritoneal and omental mucinous implants [1, 7]. A mucinous epithelial neoplasm of the appendix is the most common primary tumor. As the tumor grows to occlude the lumen, mucus accumulates and the appendix eventually ruptures. The peritoneum is then seeded with mucus-producing cells, which continue to proliferate and produce mucus. The progressive accumulation of copious amounts of mucinous material gradually fills the peritoneal cavity, resulting in the characteristic “jelly belly” [8].

Histopathological classification of pseudomyxoma peritonei is complex. A generally accepted scheme proposed by Ronnett and colleagues classifies pseudomyxoma peritonei into three subtypes with different pathological characteristics in association with different outcome: disseminated peritoneal adenomucinosis (DPAM), peritoneal mucinous carcinomatosis (PMCA), and an intermediate subtype (PMCA-I) [9, 10]. DPAM and PMCA-I were reclassified as low-grade by Bradley and colleagues [4]. They showed that the 5-year survival for PMCA was 38%, significantly worse than that for DPAM and PMCA-I (62% and 68%, respectively). These classifications are helpful to predict prognosis and guide therapy in patients with pseudomyxoma peritonei. Patients with low-grade disease are more likely to benefit from aggressive locoregional treatment, whereas those with high-grade disease should be treated as peritoneal carcinomatosis of colorectal origin [3, 10]. Irrespective of morphology, the presence of a high-grade component significantly worsens patient outcomes.

Combining cytoreductive surgery and perioperative intraperitoneal chemotherapy improves the survival of patients with pseudomyxoma peritonei [2, 11, 12]. Nonetheless, a high recurrence or progression rate of 40-70% has been reported [3]. Pathological subtype, the extent of peritoneal seeding and completeness of cytoreduction are the major determinants related to disease progression [3, 13]. Early recurrence following treatment failure is also indicative of a poor outcome [14]. Interestingly, male gender, as in our case, is associated with early recurrence [14].

At recurrence, low-grade disease typically retains its original low-grade histology. However, histological examination of recurrent or progressive disease may reveal a change in histopathological characteristics and biologic behavior. Pathological dedifferentiation is defined as a difference in pathology between the primary lesion and recurrent/progressive disease from DPAM towards PMCA-I or PMCA [3, 15]. Percentages of different histology in comparison with the initial examination are described in the range of 16% to 23% of patients [3, 8, 12]. Chua and associates reported a rate of 16% for pathological dedifferentiation in pseudomyxoma peritonei and a significant impact of this phenomenon on survival [5]. Yan and colleagues reported a series of 46 patients with pseudomyxoma peritonei who underwent cytoreductive surgery followed by a second-look operation [15]. They noted that pathological dedifferentiation occurred in 17% of patients between the first cytoreduction and the second-look operation. On the other hand, Raghav et al demonstrated that the discrepancy may result from inadequate tissue sampling [6].

Although these differences may be explained by an initial incorrect histopathological diagnosis, it is possible that evolution of low-grade neoplasm to high-grade carcinoma truly develops in some patients. The mechanism of this transformation is not certain. Recently, it has been shown that high-grade pseudomyxoma peritonei was associated with a higher frequency of p53 overexpression on immunohistochemistry [16]. Although p53 mutation is frequently observed in malignant tumors, its occurrence is particularly noteworthy among tumors showing plasticity and loss of differentiation characteristics, and p53 activation may promote differentiation in some cancers [17]. The process of pathological dedifferentiation is likely the result of the prolonged presence of disease which acquires p53 mutation at a later stage. A strategy to reduce this occurrence is to treat as early as possible and eradicate all peritoneal lesions through complete cytoreduction and sterilization of all peritoneal surface compartments with intraperitoneal chemotherapy [5].

In conclusion, transformation of low-grade appendiceal mucinous neoplasm with pseudomyxoma peritonei to high-grade sarcomatoid carcinoma is unusual, but can sometimes occur. The development of this dedifferentiation is associated with aggressive behavior and poor clinical outcome.
